# Predictors of Patients’ Loyalty Toward Doctors on Web-Based Health Communities: Cross-Sectional Study

**DOI:** 10.2196/14484

**Published:** 2019-09-03

**Authors:** Tailai Wu, Zhaohua Deng, Zhuo Chen, Donglan Zhang, Xiang Wu, Ruoxi Wang

**Affiliations:** 1 School of Medicine and Health Management Tongji Medical College Huazhong University of Science and Technology Wuhan China; 2 Department of Health Policy and Management, College of Public Health, University of Georgia Athens, GA United States; 3 School of Economics, Faculty of Humanities and Social Sciences, University of Nottingham Ningbo China Ningbo China

**Keywords:** medical informatics, telemedicine, patients, physicians, community network, psychological theory, social theory, health services

## Abstract

**Background:**

Web-based health communities provide means for patients to not only seek care but also to promote their relationship with doctors. However, little is known about the predictors of patients’ loyalty toward doctors in Web-based health communities.

**Objective:**

This study aimed to investigate the predictors of patients’ loyalty toward doctors in Web-based health communities.

**Methods:**

On the basis of sociotechnical systems theory and attachment theory, we propose that social factors including emotional interaction, perceived expertise, and social norm influence patients’ loyalty through their emotional attachment, whereas technical factors including sociability, personalization, and perceived security affect patients’ loyalty through functional dependence. To validate our proposed research model, we used the survey method and collected 373 valid answers. Partial least square was used to analyze the data.

**Results:**

Our empirical analysis results showed that all the social factors including emotional interaction (beta=.257, *t*_350_=2.571; *P*=.01), perceived expertise (beta=.288, *t*_350_=3.412; *P*=.001), and social norm (beta=.210, *t*_350_=2.017; *P*=.04) affect patients’ emotional attachment toward doctors significantly, whereas except sociability (beta=.110, *t*_350_=1.152; *P*=.25), technical factors such as personalization (beta=.242, *t*_350_=2.228; *P*=.03) and perceived security (beta=.328, *t*_350_=3.438; *P*=.001) impact functional dependence significantly. Considering the effect of working mechanisms, both emotional attachment (beta=.443, *t*_350_=4.518; *P*<.001) and functional dependence (beta=.303, *t*_350_=2.672; *P*=.008) influence patients’ loyalty toward doctors in Web-based health communities significantly.

**Conclusions:**

Patients’ loyalty toward doctors in Web-based health communities is important for the effectiveness of doctors’ advice or service in Web-based health communities. The research results not only fill the gaps in the literature of the patient-doctor relationship and Web-based health communities but also has many implications for establishing patients’ loyalty on Web-based health communities and in physical context.

## Introduction

### Background

Many people use the internet to search for health-related information and use online health services. According to a report from the Pew Research Center, almost two-thirds of US adults seek health information online, and one-third of US adults self-diagnose using information from the internet [1]. Meanwhile, many Chinese consumers have used health services online with the online health market estimated to reach 90 billion RMB in 2020 [2]. Therefore, electronic health has become an important means for people to seek care. Among electronic health applications, Web-based health communities have become increasingly important with growing utilization [3].

Web-based health communities facilitate the communication between patients and doctors online [4]. Patients and doctors could discuss health-related topics in different discussion groups using communication tools embedded in Web-based health communities [5]. Therefore, Web-based health communities could change the traditional 1-way communication from doctors to patients to bidirectional communication between patients and doctors [6].

In a patient-physician relationship, patients’ loyalty toward doctors is defined as their intention to revisit the doctor [7]. It has been shown to correlate with patients’ compliance with doctors’ medical advice, great use of medical services, and disclosure of medical information by the patient for doctors’ diagnosis [8]. Therefore, it is critical to explore the predictors of patients’ loyalty toward doctors. Previous literature has discussed this topic. For example, Rundle-Thiele and Russell-Bennett [9] found age and frequency of visits had significant effects on patients’ loyalty through satisfaction. Sutharjana et al [10] showed that the effect of organizational citizen behaviors and service quality influenced patients’ loyalty. Wu [11] demonstrated that hospital brand image had both direct and indirect effects on patients’ loyalty through service quality and patient satisfaction. Platonova et al [12] concluded that patients’ satisfaction, trust, and interpersonal relationship significantly affected patients’ loyalty toward doctors. However, few studies have considered patients’ loyalty in a Web-based health community context and studied the effect of technical environment on the formation of patients’ loyalty. Meanwhile, although previous literature studied users’ activities on the Web-based health communities, such as knowledge generation [13], knowledge sharing [14], and personal health information communication [15], few of them investigated patients’ loyalty toward doctors in Web-based health communities. Therefore, we propose our research question *What are the predictors of patients’ loyalty toward doctors in Web-based health communities?*

The remainder of this paper is organized as follows: we explore the predictors of patients’ loyalty toward doctors in Web-based health communities using attachment theory and sociotechnical system theory. Our research model hypothesizes a causal effect of the explored predictors on patients’ loyalty toward doctors in Web-based health communities. Then, consumer survey is used to collect data and validate the research model. The analysis results are discussed, and the implications are provided. The last section discusses the limitations of this work and future research directions.

### Theoretical Foundation

To examine the research questions, we used sociotechnical systems theory to explore the predictors of patients’ loyalty toward doctors in Web-based health communities. Meanwhile, to understand the working mechanisms of these predictors, we applied attachment theory to locate the mediating constructs for the predictors.

Sociotechnical systems theory was originally used to understand the organizational outcomes by dividing organizational systems into social subsystems and technical subsystems [16]. Social subsystems are mainly about people and their attributes such as values, attitudes, and skills. Meanwhile, technical subsystems concern the processes, tasks, and technologies to produce desired organizational outcomes. Therefore, the organizational outcomes are the results of the interaction between social subsystems and technical subsystems. The organizationally designated outcomes may come from the successful fit between the 2 subsystems [17]. Given that patients and doctors could constitute temporal discussion groups on Web-based health communities, patients’ loyalty toward doctors in Web-based health communities could be the outcome of patient-doctor group interaction, and we could leverage sociotechnical systems theory to understand the drivers of patients’ loyalty toward doctors on the Web-based health communities. On the Web-based health communities, the technical attributes could correspond to the technical subsystems, whereas the interaction between patients and doctors becomes the source of social subsystems. Therefore, we propose that emotional interaction between patients and doctors, perceived expertise of doctors, and social norm for patients are the social factors based on the social systems, whereas sociability, personalization, and perceived security of Web-based health communities are the technical factors according to the technical systems.

Attachment theory initially explained the reason why infants attach to their caregivers. This theory states that the attachment is described as the emotional-laden bond between infants and caregivers [18]. When the attachment is impaired, infants will be distressed or in anxiety. Several studies expanded the use of attachment theory to understand the relationship between product and consumers [19], brand and consumers [20], technology and consumers [21], and patients and providers [22]. As patients’ loyalty toward doctors in Web-based health communities is the result of the physician-patient relationship, we can use attachment theory to understand the influence of predictors of patients’ loyalty [12]. To be specific, we speculate that patients’ emotional attachment and functional dependence on doctors in Web-based health communities impact patients’ loyalty toward doctors in Web-based health communities.

On the basis of the above theories, we formulate our proposed relationships in a research model and develop corresponding hypotheses in the following section.

### Research Model and Hypotheses

**Figure 1:**
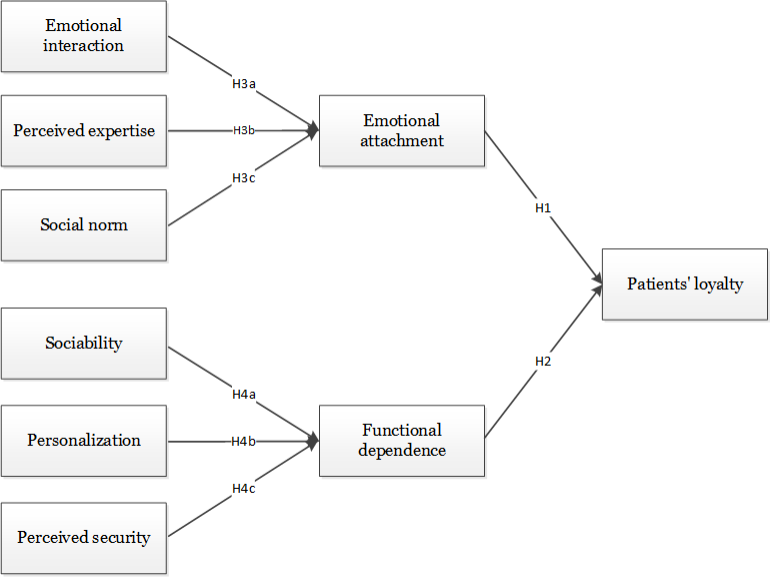
Research model.

In line with the above reasoning, we hypothesize that emotional interaction between patients and doctors, perceived expertise of doctors, and social norm for patients influence patients’ loyalty through emotional attachment, whereas sociability, personalization, and perceived security of Web-based health communities affect patients’ loyalty through functional dependence. The research model is presented in Figure 1.

### Emotional Attachment and Patients’ Loyalty

Patients’ emotional attachment to doctors in Web-based health communities is patients’ emotional bond with doctors in Web-based health communities [23]. Patients’ emotional attachment to doctors predicts patients’ commitment toward doctors [24]. Once a patient commits to a doctor, he visits the doctor persistently. Then, patients’ emotional attachment to doctors may make them revisit the doctors. Therefore, we hypothesize as follows:

H1: Patients’ emotional attachment to doctors positively influences patients’ loyalty.

### Functional Dependence and Patients’ Loyalty

Patients’ functional dependence on doctors in Web-based health communities is the interdependence between patients and doctors for solving patients’ health problems [21]. The interdependence between patients and doctors can also increase patients’ commitment to doctors [25]. The increased commitment to doctors leads a patient to revisit the doctor. Therefore, we hypothesize as follows:

H2: Patients’ functional dependence on doctors positively influences patients’ loyalty.

### Social Factors and Emotional Attachment

In this study, we identify emotional interaction, perceived expertise, and social norm as social factors. Toward patients’ emotional interaction with doctors in Web-based health communities, it is the interaction between patients and doctors that cares about patients’ emotions [26]. Compared with the interaction that only focuses on solving patients’ health problems, an interaction that cares about patients’ emotions is more important because patients cannot judge professional treatment and their negative emotions that are caused by the diseases they are suffering needs to be cared for [27]. Meanwhile, through emotional interaction with doctors, patients may form trust toward doctors in Web-based health communities [28]. Patients’ trust toward doctors helps build their emotional attachment to the doctors [24]. Therefore, we hypothesize as follows:

H3a: Patients’ emotional interaction with doctors positively influences patients’ emotional attachment.

For patients’ perceived expertise of doctors, it is patients’ perception that the doctors are valid medical professionals [29]. Perceived expertise has been defined as 1 of the 3 dimensions of perceived credibility [29]. Given trust is a reflection of perceived credibility and one of the predictors of emotional attachment [30], we hypothesize as follows:

H3b: Patients’ perceived expertise of doctors positively influences patients’ emotional attachment.

With regard to social norm, it is patients’ perception that important others approve their interaction with doctors in Web-based health communities [31]. The approval from important others represents the identification of doctors by the social group patients belong to [32]. Patients may comply with the social norm to identify the patents. The identification of doctors in Web-based health communities can drive the patients’ attachment to doctors [33]. Therefore, we hypothesize as follows:

H3c: Social norm positively influences patients’ emotional attachment.

### Technical Factors and Functional Dependence

A total of 3 factors have also been figured out to represent the technical systems: sociability, personalization, and perceived security. These factors are all functional features of Web-based health communities. Considering sociability, it is the extent to which Web-based health communities facilitate the socialization between doctors and patients [34]. The socialization between doctors and patients underpinned by Web-based health communities can make patients feel the flow in interacting with doctors in Web-based health communities [35]. The sense of flow can give rise to patients’ dependence on doctors functionally. Therefore, we hypothesize as follows:

H4a: Sociability positively influences patients’ functional dependence.

With respect to personalization, it is the extent to which Web-based health communities reflect patients’ personal needs [36]. Different patients may have different conditions, symptoms, or treatment needs, whereas they may also have different feelings and perceptions toward their diseases based on their own experience. Web-based health communities with a high degree of personalization could satisfy different patients’ needs to help them find suitable doctors for their health problems. Through interacting with a doctor who provides effective advice and support, a patient will build trust toward the doctor and rely on them [36]. Therefore, we hypothesize as follows:

H4b: Personalization positively influences patients’ functional dependence.

As to perceived security, it is the degree to which patients believe that their personal assets, such as their private information or money, will be safe when they interact with doctors in Web-based health communities [37]. When patients perceive secureness in interacting with doctors in Web-based health communities, they perceive less risky in the interaction. The lower degree of sense of risk results in the higher trust toward the doctors [38]. Therefore, we hypothesize as follows:

H4c: Perceived security positively influences patients’ functional dependence.

## Methods

### Measurement Instrument

A consumer survey was implemented in this study to test our model. To make the measurement instrument of survey, we adapted previously validated scales in our research context. Items for patients’ loyalty were from Cyr [7]. Items for emotional attachment were adapted from Vlachos et al [24], whereas items for functional dependence were based on Wu et al [21]. Toward scales for social factors, items for emotional interaction were from Ben-Sira [39], items for perceived expertise were from Ohanian [29], and items for social norm were from Venkatesh et al [31]. With regards to scales for technical factors, items for sociability were from Animesh et al [35], items for personalization were from Zhou et al [40], and items for perceived security were from Chang and Chen [41]. We used the 5-point Likert scale to measure the items with anchors ranging from 1 (strongly disagree) to 5 (strongly agree).

Our survey was conducted in China. As our scales were originally developed in English, we used back-translation method to translate the English scales into Chinese. To be specific, 1 bilingual investigator translated the English scales into Chinese at first, and another bilingual investigator translated the Chinese scales into English. Then, the 2 investigators compared the 2 English versions to check whether there were significant differences between them. To ensure the validity of our measurement instrument, we conducted a pilot study by interviewing 7 experts in the area of medical informatics and 16 users of Web-based health communities. After collecting comments from them, we revised and decided our questionnaire for the survey. The measurement instrument is showed in Multimedia Appendix 1.

Besides the constructs in our hypotheses, several control variables were included such as gender, age, education, length and experience of using Web-based health communities, and perceived health [42]. To explore the non-naive effect of respondents, respondents’ experience of filling online questionnaire was also considered [43].

### Data Collection

Given that China has the largest number of internet users and almost two-thirds of them search health information online, we chose Chinese Web-based health community users as our respondents [44]. We adopted the data collection service from a leading Chinese online survey site to administrate the survey. To ensure the data quality and reduce social desirability bias, we took several measures in designing the questionnaire and cleaning the collected data. For example, we added attention-trap and reverse-coded questions to decrease monomethod bias, whereas we set screening questions to make sure the answers from the right respondents. The screening questions included which Web-based health communities had been used, whether you were the registered member, and whether you had interacted with doctors on the Web-based health communities. Finally, we excluded cases with missing values or similar values for all questions. Our study procedures had been approved by the Institutional Review Board of Tongji Medical College, Huazhong University of Science and Technology (No. 2017S319).

## Results

### Demographic Information

Through 2 weeks’ survey, we obtained 373 complete and valid responses. In this sample, most of the respondents were in the age group of 30 years and older, females, with college degrees, and were familiar with using Web-based health communities. These results are reasonably consistent with the report of the China Internet Network Information Center on demographics of Chinese internet users [44]. The specific demographic information of this sample is summarized in Table 1.

**Table 1:** Demographic information.

Characteristics		Statistics, n (%)
**Age (years)**		
	18-25	67 (18)
	25-30	138 (37)
	>30	168 (45)
**Gender**		
	Male	160 (42.9)
	Female	213 (57.1)
**Education**		
	High school	8 (2.1)
	College	330 (88.5)
	Master’s degree and above	35 (9.4)
**Intensity of using Web-based health communities (hours/day)**		
	<0.5	305 (81.8)
	0.5-1	59 (15.8)
	>1	9 (2.4)
**Length of using Web-based health communities (years)**		
	<1	137 (36.7)
	1-5	228 (61.1)
	>5	8 (2.2)

### Reliability and Validity

To test the reliability and validity of our measurement instrument, we used partial least square (PLS) technique to analyze the collected data in SmartPLS 2.0.3M from SmartPLS GmbH in Germany [45]. Using PLS technique to conduct the confirmatory factor analysis, the values of indicators for reliability and validity are listed in Tables 2 and 3. As shown in Table 2, all the values of Cronbach alpha and composite reliabilities were above 0.7, indicating acceptable reliability for all constructs [46]. Meanwhile, the value of average variance extracted (AVE) of each construct was above 0.5, and loading weights for each item were above 0.7, thus confirming good convergent validity [46]. As shown in Table 3, the values in the square roots of AVEs were all greater than the interconstruct correlations, thus showing good discriminant validity [47]. Therefore, we concluded that our measurement instrument has enough reliability and validity to test the structural model of our proposed hypotheses.

**Table 2:** Construct reliability and convergent validity.

Construct	Items	Factor loadings	Composite reliability	Average variance extracted	Cronbach alpha
**Patient’s loyalty**					
	LYT1	0.7904	0.836	0.6299	.7062
	LYT2	0.755	—^a^	—	—
	LYT3	0.8336	—	—	—
**Emotional attachment**					
	EA1	0.8374	0.8676	0.6859	.771
	EA2	0.8128	—	—	—
	EA3	0.8343	—	—	—
**Functional dependence**					
	FD1	0.7761	0.8337	0.626	.7016
	FD2	0.7586	—	—	—
	FD3	0.8368	—	—	—
**Emotional interaction**					
	EI1	0.8635	0.8702	0.7703	.7027
	EI2	0.8917	—	—	—
**Perceived expertise**					
	PE1	0.7863	0.8436	0.6429	.7228
	PE2	0.7733	—	—	—
	PE3	0.8441	—	—	—
**Social norm**					
	SN1	0.8802	0.8862	0.7957	.7438
	SN2	0.9036	—	—	—
**Sociability**					
	SBY1	0.7974	0.833	0.6248	.7066
	SBY2	0.8255	—	—	—
	SBY3	0.7464	—	—	—
**Personalization**					
	PLN1	0.8905	0.8838	0.7919	.7372
	PLN2	0.8892	—	—	—
**Perceived security**					
	PS1	0.8808	0.8815	0.7881	.7313
	PS2	0.8946	—	—	—

**Table 3:** Discriminant validity.

Constructs	LYT^a^	EA^b^	FD^c^	EI^d^	PE^e^	SN^f^	SBY^g^	PLN^h^	PS^i^
LYT	0.7937^j^	—^k^	—	—	—	—	—	—	—
EA	0.5511	0.8282^j^	—	—	—	—	—	—	—
FD	0.4668	0.379	0.7912^j^	—	—	—	—	—	—
EI	0.4208	0.4427	0.3711	0.8777^j^	—	—	—	—	—
PE	0.4662	0.4254	0.3766	0.3013	0.8018^j^	—	—	—	—
SN	0.4812	0.3895	0.362	0.3832	0.257	0.892^j^	—	—	—
SBY	0.3407	0.3628	0.2317	0.3822	0.2085	0.4218	0.7904^j^	—	—
PLN	0.2677	0.168	0.3459	0.1957	0.1542	0.1212	0.2164	0.8899^j^	—
PS	0.4388	0.3564	0.4101	0.3778	0.3454	0.3177	0.211	0.2448	0.8878^j^

^a^LYT: loyalty.

^b^EA: emotional attachment.

^d^EI: emotional interaction.

^c^FD: functional dependence.

^e^PE: perceived expertise.

^f^SN: social norm.

^g^SBY: sociability.

^h^PLN: personalization.

^i^PS: perceived security.

^j^The square roots of average variance extracted.

^k^—: Not applicable

Given that we used the measurement instrument to collect data for all the constructs, the possibility of common method bias was tested. First, we checked the values of correlation coefficient among constructs in Table 3 and found that there were no pairs with very high correlation (r>0.90). Second, Harman single factor test was conducted by principal component analysis in SPSS 22.0. The first extracted factor in the unrotated solution accounted for 29.44%, which is less than 50% [48]. Finally, the marker variable technique was used to test the bias. Perceived organizational support, which is not relevant to our study theoretically, was set as the marker variable [49]. The average value of correlation coefficients between perceived organizational support and other variables was 0.196. Therefore, we rule out the common method bias in our study.

### Hypotheses Testing

**Figure 2:**
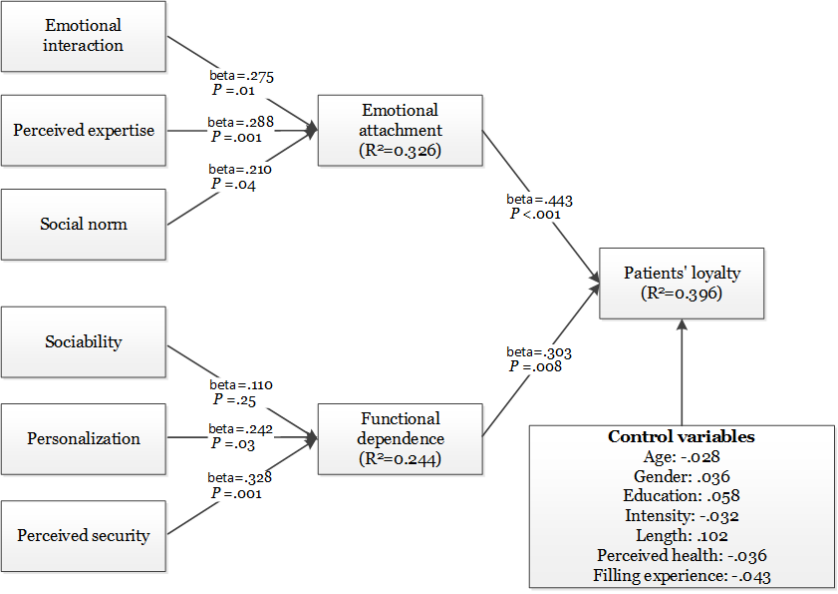
Analysis results of hypothesized model.

Through using the bootstrapping procedures in PLS, we tested the hypothesized relationships in our research model by computing the *t* values of each path. The analysis results are listed in Figure 2. According to Figure 2, both emotional attachment (beta=.443; *P*<.001) and functional dependence (beta=.303; *P*=.008) affected patients’ loyalty toward doctors in Web-based health communities significantly. Therefore, both H1 and H2 were supported. These results suggested that attachment theory provides a useful theoretical perspective to understand patients’ loyalty. Toward the effect of social factors, emotional interaction (beta=.275; *P*=.01), perceived expertise (beta=.288; *P*=.001) and social norm (beta=.210; *P*=.04) all influenced emotional attachment significantly. Therefore, H3a, H3b, and H3c were all supported. These results suggested that factors in social systems are important for the formation of emotional attachment. With regards to the effect of technical system, sociability (beta=.110; *P*=.25) did not have significant influence on functional dependence, whereas personalization (beta=.242; *P*=.03) and perceived security (beta=.328; *P*=.001) had significant influence on functional dependence. Therefore, we cannot reject H4b and H4c, whereas H4a was not supported. These results reveal that factors in technical systems are also important for functional dependence. Besides, none of the control variables had a statistically significant effect on patients’ loyalty. We think our findings are sound and strong for the following reasons: first, our measurement instrument is tested reliable and valid, and the effect of common method bias is insignificant. Second, the quality of data collection is guaranteed because several measures such as screening questions and attention-trap and reverse-rode questions are used in the questionnaires.

## Discussion

### Principal Findings

In this paper, we investigated patients’ loyalty toward doctors in Web-based health communities based on attachment theory and sociotechnical systems theory. According to sociotechnical systems, we proposed emotional interaction, perceived expertise, and social norm can represent the social systems, which lead to patients’ loyalty, whereas sociability, personalization, and perceived security comprise the technical systems, which help the formation of patients’ loyalty. Meanwhile, to explore the working mechanisms of these predictors, we leveraged attachment theory. On the basis of attachment theory, we proposed that patients’ emotional attachment corresponds to social factors and functional dependence link to technical factors. All hypothesized relationships were confirmed, except the relationship between sociability and functional dependence.

Among our proposed predictors, perceived security is shown to have the greatest impact on functional dependence, whereas perceived expertise seems to have the greatest impact on emotional attachment. This result uncovers that perceived expertise of doctors and perceived security of using the Web-based health platform are the most concerning factors when patients decide to interact with doctors in Web-based health platforms.

For the insignificant effect of sociability on functional dependence, the possible reason is that the purpose of interaction between patients and doctors is single, which is to solve consumers’ health problems to some degree. Therefore, patients may not pay attention to social tools on Web-based health communities and only use few tools to interact and communicate with doctors.

### Implications

This study has important theoretical implications. First, the application of the theoretical model on survey data provides a better understanding of patients’ loyalty to doctors in Web-based health communities. Second, this study contributes to the patient-doctor relationship literature by studying the Web-based health community context. Although previous literature about patient-doctor relationship has also studied patients’ loyalty, they study it mostly in the offline context while considering less about the influence of a Web-based health community, which is becoming a more important application in the field of digital health. Finally, this study contributes to the Web-based health community literature by studying patients’ loyalty. Although previous literature about Web-based health communities also studied patients’ activities, few have examined patients’ loyalty. Patients’ loyalty reflects the relationship between patients and doctors, which may determine the development of Web-based health communities.

Meanwhile, this study also has important implications for practice. First, this study suggests that Web-based health communities could be an important channel to improve the patient-physician relationship. Therefore, health policy makers may encourage the use of Web-based health communities. Second, predictors that represent both social and technical systems could be the basis to develop many actionable strategies to increase patients’ loyalty for clinical practice. For example, based on social factors including emotional interaction, perceived expertise, and social norm, doctors could provide more emotional support to patients, manifesting their expertise by displaying their qualification certificates and collaborate with patient group leaders to organize their virtual relationships with patients. Meanwhile, based on technical factors including personalization and perceived security, designers and managers of the Web-based health communities should understand patients’ needs to provide personalized functions and services and protect private assets including patient information and budget. These strategies can also be used by doctors, managers, and policy makers in offline context to strengthen patients’ loyalty. Finally, given that emotional attachment and functional dependence have significant influence on patients’ loyalty, the measurement scales of emotional attachment and functional dependence could be effective indicators for the formation of patients’ loyalty.

### Limitation and Future Research Direction

We point to several future research directions based on the limitations of this study. First, a technical factor–sociability does not have significant influence on functional dependence. Therefore, more factors can be explored to improve the explanatory power of this empirical model. Second, we only investigated Chinese Web-based health community users. Chinese patients’ attitudes, beliefs, and values about physician may be different from patients elsewhere [50]. Therefore, Chinese patients may behave differently when they interact with physicians. Meanwhile, culture could influence several consequences of patient-physician relationship such as satisfaction, accessibility, and continuity of care [51]. Future studies could validate this model by examining the patient-physician relationship in other cultural contexts. Finally, we used the cross-sectional data for our research model and ignored the time change effect of variables in our model. Future research can consider using the longitudinal data to capture the dynamics of our model.

### Conclusions

This study explores the predictors of patients’ loyalty toward doctors in Web-based health communities based on sociotechnical systems theory and attachment theory. Several social and technical factors are revealed, and their working mechanisms are confirmed in our empirical study. This study not only provides direct guidance to establish patients’ loyalty in the Web-based health community context but also conveys the implications for building patients’ loyalty physically.

## Appendix

Multimedia Appendix 1 Survey instrument.

## Abbreviations

AVE: average variance extracted
EA: emotional attachment
EI: emotional interaction
FD: functional dependence
LYT: loyalty
PE: perceived expertise
PLN: personalization
PLS: partial least square
PS: perceived security
SBY: sociability
SN: social norm
