# Correction: Predictors of Patients’ Loyalty Toward Doctors on Web-Based Health Communities: Cross-Sectional Study

**DOI:** 10.2196/16942

**Published:** 2019-11-07

**Authors:** Tailai Wu, Zhuo Chen, Donglan Zhang, Xiang Wu, Ruoxi Wang

**Affiliations:** 1 School of Medicine and Health Management Tongji Medical College Huazhong University of Science and Technology Wuhan China; 2 Department of Health Policy and Management, College of Public Health, University of Georgia Athens, GA United States; 3 School of Economics, Faculty of Humanities and Social Sciences, University of Nottingham Ningbo China Ningbo China

In “Predictors of Patients’ Loyalty Toward Doctors on Web-Based Health Communities: Cross-Sectional Study” by Wu et al (J Med Internet Res 2019;21(9):e14484), a minor error in the typesetting stage of publication resulted in the Acknowledgments section not being included in the final version of the article.

The Acknowledgments section has been added to the paper and appears as follows:

This work was partially supported by grants from the National Natural Science Foundation of China (No 71801100, 71671073, 71602063, 71704059, 71701075, and 71810107003).

The correction will appear in the online version of the paper on the JMIR website on November 7, 2019, together with the publication of this correction notice. Because this was made after submission to PubMed, PubMed Central, and other full-text repositories, the corrected article has also been resubmitted to those repositories.

