# Supplementary material for: Predictors of Patients’ Loyalty Toward Doctors on Web-Based Health Communities: Cross-Sectional Study
Source: J Med Internet Res. 2019 Sep 3;21(9):e14484. doi: 10.2196/14484 (PMC6751093; doi:10.2196/14484)
Supplement: Multimedia Appendix 1 [file jmir_v21i9e14484_app1.pdf]

Table A. Measurement instrument

Constructs	Items
Patients' loyalty	I would revisit this doctor on online health community.
	I would recommend this doctor on online health community to my friends.
	I would pay the services from this doctor on online health community.
Emotional attachment	I would like to be friends with the doctor on online health community.
	I would like to interact with the doctor on online health community in the future.
	I am interested in learning more about the doctor on online health community.
Functional dependence	To solve my health problems, the doctor on online health community provide resources.
	To solve my health problems or concerns, the doctor on online health community provide supports.
	To solve my health problems or concerns, the doctor on online health community provide outputs
Emotional interaction	During my interaction with this doctor on online health community, the doctor shows their interest in my problems/concerns.
	During my interaction with this doctor in online health community, the doctors give me sufficient time to present and discuss my problems.
	During my interaction with this doctor on online health community, the doctor demonstrate sufficient devotion to the management of my problems.
Perceived expertise	The doctor on online health community is an expert.
	The doctor on online health community is experienced.
	The doctor on online health community is knowledgeable
	The doctor on online health community is qualified
	The doctor on online health community is skilled.
Social norm	People who influence my behavior think that I should interact with the doctor on online health community.
	People who are important to me think that I should interact with the doctors on online health community

Sociability	Online health community enable me to get a good impression of others
	Online health community enable me to develop good social relationships with other online health community users.
	Online health community enables me to form close friendships with other online health community users.
Personalization	I “set up” the online health community to use it the way I want to.
	I have adapted online health community to meet my needs.
	I have chosen features offered by online health community to suit my style of use
Perceived security	In general, I feel secure in using this online health community.
	The online health community I used have implement security measures to protect their users.
	I would feel totally safe providing information about myself through online health community.
